# Aortic Valve Annular Characteristics in Isolated Left Ventricular Non-Compaction—Detailed Analysis from the Three-Dimensional Speckle Tracking Echocardiographic MAGYAR-Path Study

**DOI:** 10.3390/jcm14165778

**Published:** 2025-08-15

**Authors:** Attila Nemes, Nóra Ambrus, Máté Vámos, Rita B. Gagyi, Tamás Szili-Török, Zoltán Ruzsa, Csaba Lengyel

**Affiliations:** Department of Medicine, Albert Szent-Györgyi Medical School, University of Szeged, H-6725 Szeged, Hungary; ambrusnora@gmail.com (N.A.); gagyi.rita@gmail.com (R.B.G.); szili-torok.tamas@med.u-szeged.hu (T.S.-T.); ruzsa.zoltan@med.u-szeged.hu (Z.R.); lengyel.csaba@med.u-szeged.hu (C.L.)

**Keywords:** aortic valve annulus, three-dimensional, echocardiography, left ventricular, non-compaction

## Abstract

**Background:** Left ventricular (LV) non-compaction (NC) is a rare ventricular phenotype characterized by a thin compacted epicardial layer and an extensive non-compacted endocardial layer with prominent LV trabeculations and deep intertrabecular recesses. According to the recent literature, no information is available regarding the abnormalities of the aortic valve annulus (AVA) in LVNC. Therefore, the aim of the present study was to perform a detailed analysis of the AVA by three-dimensional speckle-tracking echocardiography (3DSTE) in LVNC patients and to compare the findings with matched healthy controls. **Methods:** The present study comprised 21 isolated LVNC patients, from which 9 cases were excluded due to inferior image quality. The remaining group consisted of 12 patients with isolated LVNC (mean age: 54.6 ± 13.6 years, 7 males). Jenni’s echocardiography criteria served as a definition of LVNC. The 12 patients’ results were compared to 38 healthy age- and gender-matched controls (mean age: 48.2 ± 8.0 years, 19 males). Subgroups of patients having a greater end-diastolic versus end-systolic AVA area were also compared. **Results:** Most of AVA dimensions did not differ significantly between LVNC patients and controls; however, most LVNC patients showed a larger end-diastolic AVA area (9 out of 12, 75%), which was a significantly larger ratio as seen in matched controls (11 out of 38, 29%, *p* < 0.05). Aortic valve annular plane systolic excursion (AAPSE) proved to be significantly reduced in all LVNC patients (1.12 ± 0.24 cm vs. 0.78 ± 0.28 cm, *p* < 0.05) and in LVNC subjects with a greater end-diastolic AVA area (1.11 ± 0.21 cm vs. 0.72 ± 0.21 cm, *p* < 0.05). Basal LV radial (RS) and longitudinal (LS) strains were reduced in healthy adults with a greater end-diastolic AVA area as compared to cases with a greater end-systolic AVA area. In LVNC, not only basal LV-RS and LV-LS, but also LV circumferential strain (CS) proved to be reduced regardless of whether the AVA was greater in end-diastole or in end-systole. **Conclusions:** In patients with isolated LVNC, the AVA is not dilated; however, the presence of a greater end-diastolic AVA area is observed more frequently than in healthy controls. AAPSE and basal LV-RS, LV-LS and LV-CS values are significantly reduced in LVNC irrespective of whether the end-systolic or end-diastolic AVA area is greater.

## 1. Introduction

Left ventricular (LV) non-compaction (NC) is a rare ventricular phenotype characterized by a thin, compacted epicardial layer and an extensive non-compacted endocardial layer with prominent LV trabeculations and deep intertrabecular recesses [1,2,3,4,5,6]. Several abnormalities in myocardial mechanics together with valvular and vascular changes have been described in LVNC [7]. However, according to the recent literature, no information is available regarding the abnormalities of the aortic valve annulus (AVA) and their relationship with regional LV contractility in LVNC. Advanced echocardiographic techniques like three-dimensional (3D) speckle-tracking echocardiography (3DSTE) allows detailed 3D assessment of AVA dimensions and regional LV functional properties represented by strains at the same time [8,9,10,11,12]. Therefore, this study purposed to provide a detailed analysis of the AVA and basal LV strains by 3DSTE in LVNC patients. It also aimed to compare the results with those of healthy matched controls.

## 2. Methods

**Patient population.** This study consisted of 21 patients with isolated LVNC, but 9 subjects had to be excluded due to suboptimal image quality and an inability to measure AVA dimensions. The remaining group comprised 12 cases with a mean age of 54.6 ± 13.6 years (7 males). For the diagnosis of LVNC, several echocardiographic and magnetic resonance imaging-derived criteria are available and are used clinically [3,13,14,15,16,17,18]. In the present study, diagnosis was established using one of most used Jenni’s echocardiographic criteria [3]:

− Thickened LV wall showing a two-layered structure with an epicardial compacted layer and a so-called endocardial non-compacted layer including deep intertrabecular recesses and prominent trabeculations.
− A non-compacted to compacted LV myocardial thickness ratio >2 at end-diastole, measured at the moment of maximal thickness.
− Deep intertrabecular recesses having communication with the LV cavity confirmed by color Doppler.
− Absence of concomitant cardiac anomalies.

The findings of LVNC patients were compared to those of 38 age- and gender-matched healthy controls (48.2 ± 8.0 years, 19 males) without known disorders or states potentially affecting the findings. In all individuals, complete two-dimensional (2D) Doppler echocardiography was established together with 3DSTE-derived data acquisitions. Detailed 3DSTE analysis was carried out at a later date offline. The present study is part of the **M**otion **A**nalysis of the heart and **G**reat vessels b**Y** three-dimension**A**l speckle-t**R**acking echocardiography in **Path**ological cases **(MAGYAR-Path) Study,** the purpose of which is to provide a detailed functional analysis of cardiac chambers in certain pathologies, among others (‘Magyar’ means ‘Hungarian’ in the Hungarian language). The Institutional and Regional Biomedical Research Committee (University of Szeged) approved this study under the registration number of 71/2011 (latest approval was issued on 17 March 2025). Informed consent was given by all patients and control individuals.

**Two-dimensional Doppler echocardiography**. Routine 2D echocardiographic investigation with Doppler assessment included measurement of left atrial (LA) and LV dimensions, volumes and Simpson’s ejection fraction (EF) and Doppler-based analysis of LV diastolic function (determination of E/A ratio) [19]. Larger than grade 2 valvular regurgitation and significant valvular stenosis were excluded by color Doppler imaging and measured pressure gradients, respectively [20,21]. The number of non-compacted segments was used as a characteristic of the extent of LV non-compaction by using the 16-segment LV model. For the above-mentioned purposes, a Toshiba Artida^TM^ echocardiography machine (Toshiba Medical Systems, Tokyo, Japan) with a 1–5 MHz PST-30BT phased-array transducer was applied.

**Three-dimensional speckle-tracking echocardiography**. 3DSTE studies were performed according to recent practices using the same cardiac ultrasound equipment: the Toshiba Artida^TM^ echocardiography machine (Toshiba Medical Systems, Tokyo, Japan) with a 3D-capable PST-25SX matrix-array transducer [8,9,10,11,12]. **E**nd-diastole and end-systole were defined based on electrical activity monitored by ECG. 3DSTE was conducted in 2 parts. As a first step, 3D echocardiographic data acquisitions were performed from the apical window following image optimalizations (gain, magnitude, etc.). For optimal images, 6 subvolumes during 6 heart cycles were acquired during breath-hold with constant RR intervals on the electrocardiogram (ECG). The subvolumes were stitched together by the software automatically. As a second step, vendor-specific software (3D Wall Motion Tracking, version 2.7, UltraExtend, Toshiba Medical Systems, Tokyo, Japan) was used for detailed analysis of acquired datasets.

**Figure 1:**
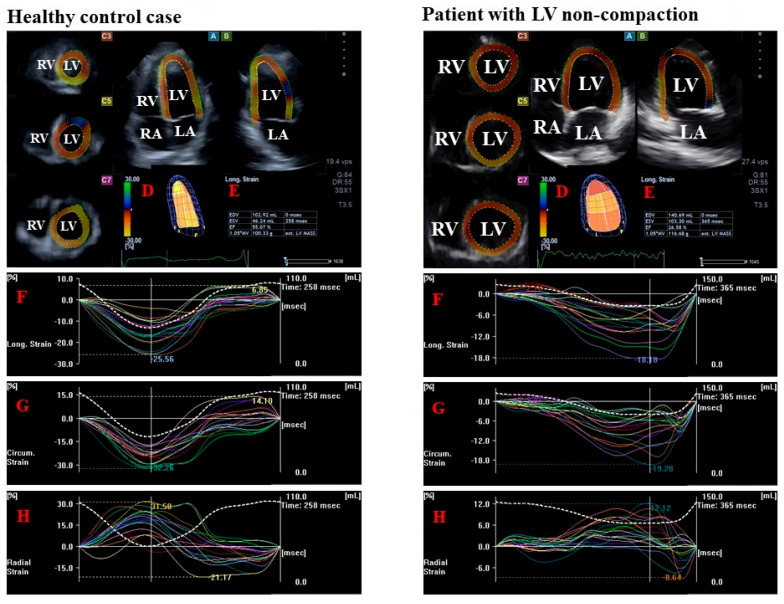
Measurement of left ventricular (LV) strains by three-dimensional (3D) speckle-tracking echocardiography in a healthy control case (**left**) and in a patient with LV non-compaction (**right**): apical longitudinal four-chamber long-axis (H) view (A) and two-chamber long-axis view (B) and short-axis views at apical (C3), midventricular (C5) and basal LV levels (C7) are presented with a virtual 3D LV cast (D) and LV volumes and ejection fraction (E). Global (white line) and segmental (colored lines) time—longitudinal (F), circumferential (G) and radial LV strain curves are presented with a time—LV volume changes curve (dotted white line). LV, left ventricle, RV, right ventricle, LA, left atrium, RA, right atrium, EF, ejection fraction, EDV, end-diastolic volume, ESV, end-systolic volume.

For LV strain analysis, together with apical 4-chamber (AP4CH) and apical 2-chamber (AP2CH) long-axis views, 3 cross-sectional views were automatically created, followed by the determination of septal and lateral edges of the LV and mitral annulus and the endocardial apical LV surface. This was followed by an automated contour detection, a sequential analysis, and the creation of a virtual 3D LV model. Several unidirectional/unidimensional basal regional LV strains were calculated (Figure 1) [8,9,10,11,12]:

− LV radial strain (RS), which represents thickening/thinning of the LV.
− LV circumferential strain (CS), which represents narrowing/widening of the LV.
− LV longitudinal strain (LS), which represents shortening/lengthening of the LV.

**Figure 2:**
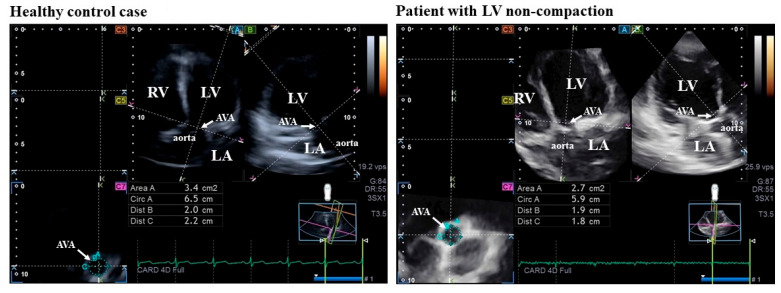
Measurement of the aortic valve annular dimensions by three-dimensional speckle-tracking echocardiography in a healthy control case (**left**) and in a patient with left ventricular (LV) non-compaction (**right**) in end-diastole (as presented on the first frame in the loop). Following optimalization of the LV longitudinal planes on apical 4-chamber long-axis view (A) and 2-chamber long-axis view (B) and visualization of the aortic valve/aorta by tilting and optimizing of the longitudinal planes in these long-axis views (on (A,B)), the planes were positioned to be parallel with the aortic root centerline. The C7 served as the cross-sectional view of the aortic valve annulus, which was perpendicular to the longitudinal plane. White yellow represents aortic valve annulus. AVA = aortic valve annulus, Area = AVA area, Circ = AVA perimeter, Dist B and C = AVA diameters, LV, left ventricle, RV, right ventricle, LA, left atrium.

For AVA dimensions, optimal LV longitudinal planes were determined on AP4CH and AP2CH views. Following visualization of the aortic valve/aorta by tilting and optimizing the longitudinal planes in AP4CH and AP2CH views, planes were positioned parallel to the aortic root centerline [12]. The C7 served as the cross-sectional view of the AVA, which was perpendicular to the longitudinal plane, allowing us to find the optimal ‘en-face’ image of the AVA. Special attention had to be paid to ensure that C7 was truly perpendicular and that measurements were not taken at the level of the Valsalva or the LV outflow tract. Several AVA characteristics have been measured in end-diastole and end-systole including minimum and maximum AVA diameter (AVA-Dmin and AVA-Dmax, respectively), AVA area (AVA-A) and AVA perimeter (AVA-P) [12]. Linear measurements were taken according to the inner edge to inner edge measurement technique, while area and perimeter data were from planimetric assessments. All these parameters were obtained in the same frame at end-diastole and end-systole, as synchronized by the ECG (Figure 2). The spatial displacement of the AVA during the cardiac cycle can be characterized by the AVA plane systolic excursion (AAPSE). In this case, after determining the plane of the AVA in end-diastole, its plane/location in end-systole was also determined, and then the AAPSE was defined as the distance between the end-diastolic and end-systolic AVA planes/locations. End-diastole and end-systole were defined as R-peak and end of T wave on ECG, respectively.

**Statistical analysis.** Data were presented in mean ± standard deviation (SD) or *n* (%) forms as appropriate. The homogeneity of variances was determined by Levene’s test. The Shapiro–Wilk test was used to test normality of distribution. In the presence of normally distributed datasets, independent samples *t*-tests were applied. In the presence of non-normally distributed datasets, the Mann–Whitney–Wilcoxon test was used. For multiple comparisons, one-way analysis of variance (ANOVA) with Bonferroni correction was applied. For categorical variables, Fisher’s exact test was applied. The Bland-Altman method was used for intra- and interobserver agreements. Reproducibility of 3DSTE-derived assessment of AVA dimensions was evaluated by two measurements of the same observer and measurements by two observers in 35 healthy individuals together with the respective interclass correlation coefficients (ICCs). A *p*-value < 0.05 was considered statistically significant. Analyses were conducted using SPSS version 29.0.0.0 (SPSS Inc, Chicago, IL, USA).

## 3. Results

**Clinical data.** Clinical data of isolated LVNC patients and healthy controls are presented in Table 1. Hypertension and hypercholesterolaemia were frequent in isolated LVNC patients. Age of LVNC patients with vs. without hypertension did not differ (56.2 ± 14.0 years vs. 52.7 ± 12.9 years, *p* = ns) with similar ratios of use of angiotensin-converting enzyme (80% vs. 71%, *p* = 0.85), beta-blockers (80% vs. 71%, *p* = 0.85) and diuretics (80% vs. 71%, *p* = 0.85). The mean duration of hypertension proved to be 20 ± 5 years. None of the LVNC patients showed signs of significant hypertrabeculation of the right ventricle.

**Two-dimensional Doppler echocardiographic data.** Dilated 2D echocardiography-derived LA and LV sizes and volumes with impaired LV-EF could be demonstrated in patients with isolated LVNC as compared to matched healthy controls. The mean number of non-compacted segments was 6.4 ± 1.5. Grade 1 and 2 mitral regurgitation (MR) could be demonstrated in four and four isolated LVNC patients, respectively. Grade 1 and 2 tricuspid regurgitation (TR) could be detected in three and one isolated LVNC patients, respectively. A higher grade of MR and TR was not present. No patient with isolated LVNC showed equal/larger than grade 1 aortic regurgitation. None of the isolated LVNC patients and healthy controls showed significant valvular stenosis. Only 6 out of 72 basal LV segments (8%) proved to be non-compacted in LVNC patients.

**Three-dimensional speckle-tracking echocardiographic data.** The average frame rate for 3DSTE was 31 ± 2 fps, which proved to be similar between controls and LVNC patients. Most LVNC patients showed a larger end-diastolic AVA-A (9 out of 12, 75%), which was a significantly larger ratio as seen in matched controls (11 out of 38, 29%, *p* < 0.05). The remaining healthy controls had mostly larger ratio of end-systolic AVA-A (23 out of 38, 61%) or the end-diastolic and end-systolic AVA-As proved to be the same sized (4 out of 38, 11%). The remaining 3 LVNC cases had larger end-systolic AVA-A (3 out of 12, 25%). No significant differences were found in AVA dimensions between LVNC patients and controls, except for end-systolic AVA-Dmin and AVA-A, which proved to be larger in healthy controls. End-diastolic and end-systolic AVA-Dmin were larger in healthy cases vs. LVNC patients with a greater end-diastolic AVA-A. AAPSE proved to be significantly reduced in all LVNC patients and in LVNC subjects with a greater end-diastolic AVA-A. Basal LV-RS and LV-LS were reduced in controls with a greater end-diastolic AVA-A versus controls with a greater end-systolic AVA-A, which was associated with greater end-diastolic AVA dimensions. Regional basal LV functions represented by LV-RS, LV-CS and LV-LS proved to be similarly significantly reduced in all LVNC patients and in both subgroups compared to that of controls; however, no significant differences were observed in these strain values between the LVNC subgroups. Duration of hypertension and age did not correlate with any AVA parameters in LVNC (Table 2).

**Reproducibility of 3DSTE-derived AVA assessments.** Intraobserver and interobserver agreements of 3DSTE-derived end-systolic and end-diastolic AVA maximum and minimum diameters, AVA areas and AVA perimeters, along with the respective ICCs, are demonstrated in Table 3.

## 4. Discussion

According to the recent 2023 guidelines of the European Society of Cardiology, LVNC is not considered to be a cardiomyopathy but instead a phenotypic trait, and the term ‘hypertrabeculation’ is recommended instead of LVNC [22]. However, LVNC was considered to be a rare cardiomyopathy previously [23], and significant literature data demonstrate that myocardial mechanics and valvular and vascular functions show abnormalities in a series of LVNC patients [7]. Regarding mitral (MA) and tricuspid annular (TA) morphology and function, both showed dilation in the presence of isolated LVNC with impaired sphincter-like function for MA and preserved sphincter-like function for TA [7]. A similar analysis for AVA, however, has never been performed before.

Cardiovascular imaging like 3DSTE has undergone a significant development in the last decades. One of the main advantages of 3DSTE in addition to its non-invasive and easy-to-learn/easy-to-perform nature is that not only all chambers but also the valves and their annuli can be assessed in detail at the same time using the same acquired 3D echocardiographic datasets [8,9,10,11,12]. 3DSTE has been validated for LV volume and strain assessments [24,25,26,27], and its usefulness for AVA assessment has also been confirmed [12]. Therefore, 3DSTE enables (patho)physiologic studies. Although several studies confirmed significant abnormalities of LV volumes and functional properties in LVNC, its relationship with AVA dimensions and AAPSE has never been examined [7]. During the literature search, using ‘non-compaction’/‘noncompaction’ and ‘aortic valve’ terms, no studies were found suitable to this research.

The LV, the aortic valve and the aorta form an organic morphological and functional unit (ventricular/valvular/vascular coupling). The aortic valve is located between the ascending aorta and the LV, having three crown-shaped thin leaflets forming a semilunar pattern and interleaflet triangles. It has a fibrous annulus (AVA) as well, enabling one-way blood flow, being open in systole and closed in diastole. AVA is not an optimally circle-shaped structure but is more oval in shape, which is confirmed by the different diameter values in different directions in most cases. Unlike those seen on the atrioventricular valves, it has been demonstrated that in appr. 60% of cases, the end-systolic AVA area is greater than the end-diastolic area [12,28,29]. The surrounding structures located subvalvularly and supravalvularly are in active contact with the valve.

When regional LV function was compared between healthy cases with a larger end-diastolic AVA-A versus end-systolic AVA-A, subjects with a larger end-diastolic AVA-A showed significantly reduced basal LV-RS, LV-LS and basal rotation [28]. According to the presented findings, in LVNC cases, a significantly higher ratio of individuals showed a larger end-diastolic AVA-A than the matched healthy controls. Regardless of whether the end-diastolic or end-systolic AVA area was greater, similar significantly reduced basal LV-RS, LV-CS and LV-LS together with decreased AAPSE could be detected in LVNC patients in the present study.

These findings could have several implementations. First of all, it has been demonstrated that assessment of AVA dimensions, AAPSE and regional LV strains can be performed at the same time not only in healthy subjects but also in a disorder like LVNC with a difficult-to-examine nature. However, it should be confirmed whether measured AVA abnormalities have any diagnostic or prognostic impact in NCCM. Secondly, in most LVNC cases, end-diastolic AVA dimensions are greater, while in healthy subjects mostly end-systolic AVA sizes are larger. Thirdly, most of AVA dimensions do not differ between LVNC cases and healthy subjects except for end-systolic AVA-Dmin and AVA-A, which are larger in controls; moreover, if end-diastolic AVA dimensions are greater than end-systolic ones, some parameters are larger in healthy subjects as compared to those of LVNC patients. Fourthly, AAPSE representing spatial displacement of AVA is reduced in LVNC regardless of which cardiac cycle AVA dimensions are greater, which can be partly explained by all LV strain values being significantly reduced, values which represent LV contractility in different directions in the 3D space. All these findings suggest reduced spatial AVA displacement due to decreased basal regional LV function without changes in AVA dimensions in LVNC. However, further studies are warranted to confirm the presented findings in a larger patient cohort with more accurate and reliable methods.

### Limitation Section

The most important limitations are listed below:

− Image quality of 2D echocardiography is still better than that of 3DSTE due to several technical reasons like better spatial and temporal resolutions. Moreover, there is a size difference between the transducers for 2D echocardiography and 3DSTE: the larger size of the 3DSTE transducer makes its positioning on the chest more complicated. Moreover, the fact that six subvolumes during six cardiac cycles are required for optimal image quality may lead to stitching and motion artefacts [8,9,10,11,12]. Only acceptable quality images were used during analysis; low-quality images or those with extreme results were excluded. If more than quarter of the LV/AVA could not be visualized, the analysis was considered infeasible.
− It is not clear whether frame rate modifies 3DSTE-derived AAPSE measurements. Moreover, 2D echocardiography-guided M-mode tracings and 3DSTE-derived assessments of the AAPSE were not compared, which could be considered as a technical limitation of the present study but could be a topic for future investigations.
− Only data of a limited number of patients with LVNC were analyzed in this study. However, it should be noted that LVNC is a rare disease. The small LVNC sample size potentially introduced selection bias. Moreover, no power calculation or justification of sample size has been provided, limiting the generalizability and power of the findings.
− Moreover, only Caucasian (Hungarian) subjects from a single center were involved, which can also be considered a limitation.
− The most important preselection bias is that due to low-quality images, 43% of LVNC patients had to be excluded, which could significantly affect the findings and should be considered when interpreting the results.
− According to literature data from the MAGYAR-Healthy Study, similarly to LVNC cases, AVA parameters could not be measured in 40% of healthy subjects due to technical reasons [12]. In the present study, age- and gender-matched healthy subjects served as controls.
− Some cardiovascular risk factors including hypertension and hypercholesterolemia were relatively frequent in patients with LVNC, which could partially explain the findings. In a recent review it has been demonstrated that a lifetime exposure to elevated systolic blood pressure may be associated with an increased risk of (aortic) valvular heart disease [30]. Similarly, hyperlipidemia was found to be associated with aortic valve disease [31].
− Although there are several opportunities, STE-derived or other parameters characterizing AVA dimensions were not intended to be investigated in this study.
− Moreover, grading of valvular regurgitation was mainly performed visually rather than with quantitative and advanced methods, limiting precision.
− The controls and LVNC patients were grouped according to their AVA-A. As can be seen from the data presented, it might have been worthwhile to also group patients according to AVA diameters and perimeter, although this would have significantly exceeded the scope of this scientific work.
− The effect of compaction on the relationship between LV function and AVA was not examined in this study due to the low number of non-compacted basal segments (8%) in LVNC patients. According to literature data, however, similarly reduced LV-LS and LV-CS of compacted and non-compacted segments in LVNC were demonstrated in a recent study. The reduction in LV-RS was more pronounced in the presence of non-compaction [32].
− Moreover, LV rotational mechanics was also not intended to be examined in the present study, which could be a topic of further analysis.

## 5. Conclusions

In patients with isolated LVNC, no AVA dilation can be detected. A greater end-diastolic AVA-A is seen more frequently in LVNC patients as compared to healthy controls. AAPSE is significantly reduced in LVNC, regardless of whether the end-diastolic or end-systolic AVA-A is larger, which can be partly explained by reduced all basal LV strains.

## Figures and Tables

**Table 1:** Clinical and two-dimensional echocardiographic data of patients with isolated left ventricular non-compaction and controls.

	Controls (*n* = 38)	Isolated LVNC Patients (*n* = 12)
**Risk factors**		
Age (years)	48.2 ± 8.0	54.6 ± 13.6
Male gender (%)	19 (50)	7 (60)
Hypertension (%)	0 (0)	5 (42) *
Diabetes mellitus (%)	0 (0)	0 (0)
Hypercholesterolemia (%)	0 (0)	3 (25) *
Basal heart rate (1/s)	78 ± 3	85 ± 8
Systolic blood pressure (mm Hg)	122 ± 5	134 ± 12
Diastolic blood pressure (mm Hg)	80 ± 3	85 ± 6
**Two-dimensional echocardiography**		
LA diameter (mm)	38.3 ± 4.5	46.0 ± 8.8 *
LV end-diastolic diameter (mm)	47.9 ± 3.8	62.7 ± 11.7 *
LV end-diastolic volume (mL)	107.4 ± 21.7	198.6 ± 79.9 *
LV end-systolic diameter (mm)	32.1 ± 3.4	48.0 ± 13.4 *
LV end-systolic volume (mL)	37.3 ± 9.5	117.4 ± 67.3 *
Interventricular septum (mm)	9.4 ± 1.2	10.2 ± 1.7
LV posterior wall (mm)	9.5 ± 1.4	9.9 ± 1.3
LV ejection fraction (%)	65.1 ± 3.9	39.1 ± 14.3 *

LVNC = left ventricular non-compaction, LA = left atrial, LV = left ventricular. * *p* < 0.05 vs. Controls.

**Table 2:** Comparison of three-dimensional speckle-tracking echocardiography-derived aortic valve annular dimensions and aortic valve annular plane systolic excursion between patients with isolated left ventricular non-compaction and controls.

	All Controls (*n* = 38)	Controls with Greater End-Diastolic AVA-A (*n* = 17)	Controls with Greater End-Systolic AVA-A (*n* = 21)	All Isolated LVNC Patients (*n* = 12)	Isolated LVNC Patients with Greater End-Diastolic AVA-A (*n* = 9)	Isolated LVNC Patients with Greater End-Systolic AVA-A (*n* = 3)
AVA-Dmax-D (cm)	2.08 ± 0.26	2.18 ± 0.28 * #	1.99 ± 0.20 *	2.04 ± 0.48	2.08 ± 0.50 *	1.93 ± 0.37
AVA-Dmin-D (cm)	1.86 ± 0.26	1.94 ± 0.23 ‡	1.79 ± 0.27 *	1.72 ± 0.27 *	1.70 ± 0.29 *	1.77 ± 0.21
AVA-A-D (cm^2^)	3.30 ± 0.78	3.55 ± 0.82 * #	3.09 ± 0.67 *	3.19 ± 1.08	3.26 ± 1.13 *	3.00 ± 0.88
AVA-P-D (cm)	6.48 ± 0.78	6.74 ± 0.79 * #	6.26 ± 0.70 *	6.33 ± 1.13	6.40 ± 1.20 *	6.13 ± 0.87
AVA-Dmax-S (cm)	2.02 ± 0.27	1.97 ± 0.28	2.06 ± 0.26	1.92 ± 0.50	1.81 ± 0.53	2.20 ± 0.22
AVA-Dmin-S (cm)	1.90 ± 0.29 †	1.86 ± 0.34 ‡	1.93 ± 0.24	1.58 ± 0.40	1.49 ± 0.39	1.83 ± 0.31
AVA-A-S (cm^2^)	3.33 ± 0.85 †	3.07 ± 0.93	3.55 ± 0.71	2.69 ± 1.04	2.37 ± 0.89	3.67 ± 0.82
AVA-P-S (cm)	6.48 ± 0.88	6.23 ± 0.94	6.69 ± 0.77	6.02 ± 1.31	5.76 ± 1.38	6.80 ± 0.70
AAPSE (cm)	1.12 ± 0.24 †	1.11 ± 0.21 ‡	1.12 ± 0.27	0.78 ± 0.28	0.72 ± 0.21	0.97 ± 0.37
Basal LV-RS (%)	31.8 ± 11.8 †	27.6 ± 8.5 # ‡	35.2 ± 12.9 @	15.0 ± 8.0	15.4 ± 8.4	13.4 ± 7.6
Basal LV-CS (%)	−26.1 ± 5.6 †	−27.2 ± 5.6 ‡	−25.2 ± 5.4 @	−10.2 ± 3.1	−10.4 ± 3.3	−8.6 ± 2.5
Basal LV-LS (%)	−20.4 ± 4.4 †	−18.9 ± 3.5 # ‡	−21.5 ± 4.6 @	−9.1 ± 2.2	−9.0 ± 2.3	−8.7 ± 2.0

LVNC = left ventricular non-compaction, AVA = aortic valve annulus, Dmax = maximum diameter, Dmin = minimum diameter, A = area, P = perimeter, D = end-diastolic, S = end-systolic, AAPSE = aortic valve annular plane systolic excursion. * *p* < 0.05 vs. end-systolic counterpart; † *p* < 0.05 vs. all isolated LVNC patients; ‡ *p* < 0.05 vs. isolated LVNC patients with greater end-diastolic AVA-A; # *p* < 0.05 vs. all controls with greater end-systolic AVA-A; @ *p* < 0.05 vs. isolated LVNC patients with greater end-systolic AVA-A.

**Table 3:** Intra- and interobserver variability for three-dimensional speckle-tracking echocardiography-derived assessment of aortic valve annular dimensions and aortic valve plane systolic excursion.

	Intraobserver Agreement		Interobserver Agreement	
	Mean ± 2SD Difference in Values Obtained by 2 Measurements of the Same Observer	Interclass Correlation Coefficient Between Measurements of the Same Observer	Mean ± 2SD Difference in Values Obtained by 2 Observers	Interclass Correlation Coefficient Between Independent Measurements of 2 Observers
**AVA-Dmax-D (cm)**	−0.05 ± 0.19	0.87 (*p* < 0.01)	−0.06 ± 0.18	0.87 (*p* < 0.01)
**AVA-Dmin-D (cm)**	−0.01 ± 0.25	0.90 (*p* < 0.01)	−0.03 ± 0.23	0.93 (*p* < 0.01)
**AVA-A-D (cm^2^)**	−0.13 ± 0.60	0.94 (*p* < 0.01)	−0.11 ± 0.58	0.95 (*p* < 0.01)
**AVA-P-D (cm)**	−0.07 ± 0.65	0.90 (*p* < 0.01)	−0.12 ± 0.70	0.95 (*p* < 0.01)
**AVA-Dmax-S (cm)**	0.01 ± 0.32	0.91 (*p* < 0.01)	0.02 ± 0.33	0.93 (*p* < 0.01)
**AVA-Dmin-S (cm)**	0.07 ± 0.34	0.81 (*p* < 0.01)	0.04 ± 0.37	0.83 (*p* < 0.01)
**AVA-A-S (cm^2^)**	0.12 ± 0.70	0.91 (*p* < 0.01)	0.13 ± 0.77	0.95 (*p* < 0.01)
**AVA-P-S (cm)**	−0.01 ± 0.58	0.91 (*p* < 0.01)	0.02 ± 0.53	0.92 (*p* < 0.01)
**AAPSE (cm)**	−0.02 ± 0.20	0.90 (*p* < 0.01)	−0.03 ± 0.20	0.90 (*p* < 0.01)

AVA = aortic valve annulus, Dmax = maximum diameter, Dmin = minimum diameter, A = area, P = perimeter, D = end-diastolic, S = end-systolic, AAPSE = aortic valve annular plane systolic excursion.

## Data Availability

The data presented in this study are available on request from the corresponding author.
